# "Well-Guessed" or "Well-Done"?

**Published:** 1887-08-20

**Authors:** 


### "WELL-GUESSED" OR "WELL-DONE"?

In his essay on "Truth-hunting," the author of *Obiter Dicta* thus speaks: "In these days of Champagne and Shoddy, of display of teacups and rotten foundations—especially, too, now that the 'nexus' of 'cash payment,' which was to bind man to man in the bonds of a common pecuniary interest, is hopelessly broken—it becomes plain that the real wants of the age are not analyses of religious belief, nor discussions as to whether 'Person' or 'Stream of Tendency' are the apter words to describe God by; but a steady supply of honest, plain-sailing men who can be safely trusted with small sums, and to do what in them lies to maintain the honour of the various professions, and to restore the credit of English workmanship. We want Lambs, not Coleridges. The verdict to be striven for is not 'Well-guessed' but 'Well-done.'"

The tendency of the times, among many medical men in this country, and in Germany and France, is to pursue medicine as a bare science, and to be ashamed of it as a practical art. The man of the laboratory and the library thinks himself a vastly superior person to him of the bedside. It has always happened that the writer of books has looked down upon the ploughman and the reaper as mere uneducated louts. The reader of those same books has generally accepted this verdict, because the ploughman, being better employed, has not taken the trouble to contradict his superior fellow-being of the library. But we venture to think that the book-shelf man would find himself quite as much nonplussed at the plough-tail as the ploughman would among the masses of witless print which constitute the furniture of many book rooms. If the world were ever called upon to weigh the writers of books against the masters of the practical arts in impartial scales, the scribes would very soon find themselves in the act of kicking the beam. The philosophy of tailoring can be very well dispensed with, but every civilised man must have trousers; and the classification of headaches, though interesting in itself, can be better done without than two grains of blue pill. It is a mere impertinence on the part of academical persons to look upon themselves as cultured, and to regard all the rest as barbarians. The practical arts are what we all live by, and are the really indispensable possessions. The truly great men of the world are those who are great in action,—the teacher, the soldier, the sailor, the surgeon, the ploughman, the backwoodsman, the carpenter, the mason, the bridge-builder, the road-maker, and a hundred other "workers." Your mere essayist, verse writer, theory propounder, critic, word-spinner,—what is he but the owner of a barrel filled with so much, more or less frothy, talk, of which barrel he can turn on the tap at convenience, to his own profit and the world's frequent bewilderment and greater confusion.

The division of labour has great advantages within limits. It is very easy to see that no one man possesses "all the talents." "Not this man nor that man, but all men, make up mankind, and their united tasks the task of mankind," says Carlyle; and it is therefore manifestly expedient that persons who show aptitudes for certain work should be put to that work, if the work itself be good and necessary. But every man should have some sense of proportion; and arts which are merely ancillary should never presume to rank themselves above that particular art to which they are handmaids, and except for the convenience of which they would never have needed to be brought into existence at all. The professor of the more or less speculative sciences of physiology and pathology will rank himself higher than the skilful physician: and the Royal Unilinear Society, or Society of one line of things, will endorse his claims, and leave his more useful fellow-ant out in the cold. But, except for the necessities of the medical art, who would have studied anatomy, physiology, or pathology? Medicine is the great-grandfather of them all, and, like veritable conceited and ill-bred youngsters, they never weary of proclaiming their superiority to the author of their own being. This is excessively silly, and shows a complete want of mental breadth and artistic perspective.

It cannot be too urgently repeated, that of all the arts and sciences which have to do with "medicine" in its broadest sense the actual practice of medicine is the highest and most important. It is eminently great in itself, and is the sole *raison d'être* of all the others. A man should not confine his vision to his own little period; still less to his own country, or city, or hospital. It is amazing, and rouses the keenest contempt, to see persons who are members of what are called Universities, seats for the teaching of all learning,—such persons wilfully and deliberately shutting themselves up, like the farmer's cock to his own particular dunghill, and crowing out with the utmost power of their lungs to all the world that their little science is the most scientific of all the sciences, and that they themselves are the most learned of all its learned professors. Miserable bipeds! It is not worth while even to wring their necks.

If two or three young men, who know nothing of the world outside the laboratory and a strictly limited and purely professional library, were the only persons who took this view of things, it would not matter. If a minnow is happy in the delusion that his little pool is the world, that hurts nobody and pleases the minnow. But there are so many stupid sheep in the human fold, that any fool who "sets up" in the trade of bell-wether is sure to have troops of followers; and so it has come to pass that many persons affect to despise the art of the mere practitioner of medicine. Even that would be of little moment were it not that people who find their work undervalued often have a kind of feeling that the general verdict must be right, and so begin to undervalue both their work and themselves, not only to their personal disadvantage, but to the great injury of their art. The general practice of medicine has suffered greatly, and still suffers from the poor esteem in which it is held in comparison with some of the sciences which are subordinate to it. We enter our most energetic protest against this sentiment of the times, and ask for a judgment which shall re-establish the noblest and most humane of all the arts and sciences upon that just eminence which belongs to it by indisputable right.

